# Gastrointestinal bleeding due to gastrointestinal lipoma: A case report

**DOI:** 10.22088/cjim.12.4.622

**Published:** 2021

**Authors:** Seifollah Rezaii, Ali Enshaii, Afshin Zahedi, Morteza Amestejani, Mohsen Herik Dizaji

**Affiliations:** 1 Department of General Surgery, Urmia University of Medical Sciences, Urmia, Iran

**Keywords:** Benign, Gastric lipoma, Hematemesis, Submucosal tumors

## Abstract

**Background:**

Lipomas are common harmless tumors that are created in the colon in the gastrointestinal tract. The aim of this study was to report a case study on gastrointestinal lipoma with GIB.

**Case Presentation:**

A 38-year-old man was admitted to the hospital because of gastrointestinal bleeding for two months in December 2017. He had abdominal pain, dyspeptic disorders, vomiting, melena, and intermittent hematemesis without significant weight loss. Upper gastrointestinal endoscopy indicated a large subepithelial lesion in the antrum of the stomach with near-normal mucosa. Endoscopic ultrasound (EUS) showed a large well-defined heterogeneous mass-like lesion in the antrum of the stomach. A subtotal gastrectomy was done in the patient. The histology results of the separated samples presented a gastric lipoma.

**Conclusion:**

Gastric lipoma is often yellowish. It might ulcerate and bleed, but it does so, only rarely. It most frequently occurs as a solitary and smooth mass in the gastric antrum.

Lipomas comprise 3% of gastrointestinal tumors with a prevailing spot in the colon (60-75%) and the small intestine (30%) (1). Lipomas are scarce and benignant submucosal tumors composed of mature fatty tissues. They grow everywhere in the gastrointestinal tract, but their occurrence is rare. Usually, most gastric lipomas are discovered coincidently (2); they can lead to drastic symptoms such as occlusion, invagination, and gastrointestinal hemorrhage (3,4). Traditionally, sonography findings suggest that a gastric lipoma is a soft, steeply marginated, oval, or spheral mass. Accordingly, it is condensable during the fluoroscopic examination and may indicate noticeably decreased debilitation on barium studies (5,6). During the endoscopy procedure, a classic yellowish, soft subepithelial pile, with recurrently styled aspects like tenting, cushion, or naked fat sign was demonstrated (1). On the other hand, the lesions are well-defined areas of uniform, fatty compression with an attenuation ranging from –70 to –120 H. Therefore, a gastric lipoma can certainly be detected using CT. If the patient is asymptomatic, the endoscopy or the surgery will be obviated (7,8). 

## Case Presentation

A 38-year-old man was admitted to the hospital because of gastrointestinal blood loss for two months in December 2017 (Ethical Code: IR.UMSU.REC.1399.360). He had abdominal pain, dyspeptic disorders, vomiting, melena, and intermittent hematemesis without significant weight loss.

His past medical history indicated no related conditions but reported a history of smoking 10- pack-years and alcohol consumption. On physical examination, his height was 178 cm, weight 124 kg (BMI: 39/2 KG/M2), blood pressure was 100/60 mmHg, and his pulse rate was 80/min. There was also no intake of nonsteroidal anti-inflammatory drugs. 

**Figure 1:**
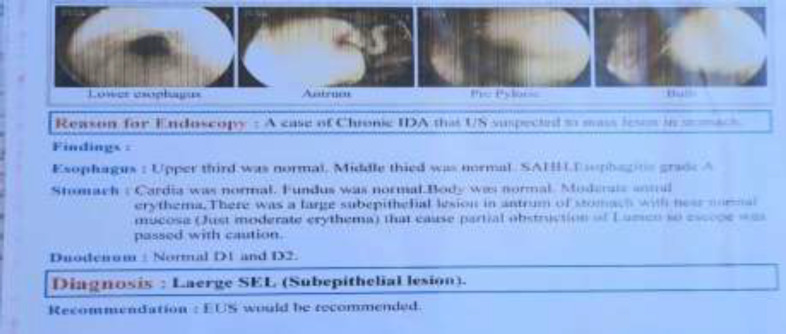
Endoscopy view: a large subepithelial lesion

**Figure 2.**
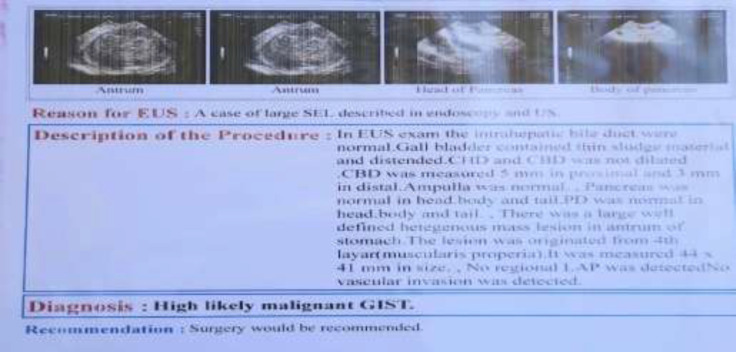
Endoscopic ultrasound findings: a large well-defined heterogeneous mass lesion

There were no unusual findings in the chest x-ray, and no icterus existed. The abdomen was noticeably full of fat, but a common intestine sound was heard. Epigastric pain was felt in the stomach. Laboratory results showed leukocytosis (WBC: 13940, PMN: 83.5%), and other test results were normal. Upper gastrointestinal endoscopy disclosed a large subepithelial lesion in the antrum of the stomach with near-normal mucosa that causes a partial obstruction of the lumen, so the scope was passed with caution (figure 1). Because of the submucosal lesion, there is no possibility of preoperative biopsy. Endoscopic ultrasound (EUS) is the most exact method in categorizing lipomas as hyperechoic lesions caused by the submucosal layer (1). In this study, endoscopic ultrasound (EUS) revealed a large well-defined heterogeneous mass lesion in the antrum of the stomach. The lesion originated from the fourth layer (muscularis propria). It was measured 44×41 mm in size. Neither peripheral lymphadenopathy (LAP) nor vascular invasion was detected (figure 2). 

According to endoscopic results, we decided to perform surgery. The patient underwent midline supraumbilical laparotomy. Intraoperatively, a yellowish, smooth surface subserosal wound with a linear diameter of around 5 cm was detected in the antrum of the stomach. Subtotal gastrectomy was performed for removing the mass with the appropriate margin. Pathology examination of specimens confirmed gastric lipoma consists of a stomach segment measuring 13×6×4 cm with two pieces of separated adipose tissue measuring 20×10×3 cm. A submucosal well-defined creamy-colored mass with the greatest dimension, measuring 7 cm, was detected with a distance of 2.5 cm from the closest surgical margin. The patient was discharged on the sixth postoperative day with a stable vital sign and appropriate feeding. 

## Discussion

Gastric lipomas are detected marginally more often in women than in men and commonly affect the population between 40 to 70 years of age (9). Lipomas are common harmless tumors that are created in the colon in the digestive tract (10). The gastric lipoma is often a yellowish substance that might become wounded and bleeds, but it happens rarely (11). Gastric lipomas are often regarded as an intramucosal mesenchymal tumor of mature adipocytes (12 13). Most of these gastric lipoma tumors are without symptoms that can be managed without treatment until confirming their diagnosis.

On the contrary, if a gastric tumor cannot be recognized as a lipoma or be in the vicinity of a hemorrhagic ulcer should be removed surgically (14 15). The occurrence of gastric lipoma is scarce, with only about 220 cases reported in the medical literature (16). Even though the etiology of gastric lipomas is unclear, according to some previous studies, it may construct a wrong embryological displacement or an acquired status (17). Gastric lipomas most commonly happen as single and soft masses in the gastric antrum (2, 3). Although most lipoma cases have no signs and are diagnosed incidentally, they can have symptoms such as abdominal pain, dyspepsia (indigestion), gastrointestinal bleeding (hemorrhage), intussusception, and bowel obstruction (intestine occlusion). These mentioned signs might relate to the size of the lipomas that endoscopic results of gastric lipomas are presented, such as cushion sign, the sponge-like sinking, the easiness of contraction with a biopsy forceps of the normal mucosa overlying the lesion, and yellowish mucosa (18, 19). It has been reported that, depending on the size, if a gastric lipoma is larger than 2 cm, it will present abdominal pain more than 50% of the time (20). 

In a study conducted by Machado et al., 37% of the cases were diagnosed with either chronic or acute gastrointestinal bleeding, obstruction, or dyspepsia. In this study, most of the large stomach lipomas that have been presented were cured by primary gastric resection (10). In another related study by Brandler in 1894, a 22.7-kg lipoma (the largest subcutaneous lipoma) was taken from the left scapula of a male patient who was 26-year old (14). However, explicit recognition of lipomas is hard and arduous work because routine biopsies generally manifest only normal gastric mucosa. By abdominal computed tomography (CT), lipomas are usually imagined and well-determined as submucosal masses with monotonous fat. 

In this case report study, a 38-year-old man presented with hematemesis, pain, and melena. So, the surgical excision was performed with subtotal gastrectomy. An intramural mass was found in the antrum, causing a degree of lumen structure. There was an ulcer on the surface of this tumor. In our case, a large intramural lipoma of the stomach with areas of reactive fibrosis, the ulcerated area of tumor with mild inflammation, and small hyperplasia of the gastric mucosa near the ulcer was recognized. The remaining gastric mucosa appears with no substantial lesions. Microcystic dilation of the gastric glands is recognized (14). 
